# Observational studies on the association of outpatient antidiabetic medication use and COVID-19 outcomes: are the findings more relevant to diabetes management than to COVID-19 pathology? A mini-review

**DOI:** 10.3389/fcdhc.2026.1760695

**Published:** 2026-04-14

**Authors:** Jelena Dimnjaković, Tamara Buble, Ognjen Brborović

**Affiliations:** 1 Medical Informatics and Biostatistics Division, Croatian Institute of Public Health, Zagreb, Croatia; 2 Department of Social Medicine and Health Care Organization, Sveuciliste u Zagrebu Medicinski fakultet, Zagreb, Croatia

**Keywords:** cardiovascular outcome, COVID - 19, diabetes mellitus, observational trials, outpatient treatment

## Abstract

At the start of the COVID-19 pandemic, there were concerns that some antidiabetic medications might worsen outcomes, though anti-inflammatory properties suggested possible benefits. Many observational studies examined antidiabetic medications use and COVID-19 outcomes. Meta-analyses showed that insulin was linked to worse outcomes, while metformin, sodium-glucose cotransporter 2 (SGLT-2) inhibitors, and glucagon-like peptide-1 (GLP-1) agonists were associated with better outcomes. Findings on dipeptidyl peptidase-4 (DPP-4) inhibitors, pioglitazone, and sulfonylureas were mixed—showing neutral, beneficial, or negative effects. However, randomized controlled trials (RCTs) testing these medications after SARS-CoV-2 infection found no effect on COVID-19 outcomes, implying that their anti-inflammatory effects do not translate into meaningful clinical benefits during acute infection. This discrepancy prompts questioning what observational studies actually measured. Given that many studies applied robust statistical methods, their results are unlikely solely due to confounding or indication bias. We hypothesize that these studies reveal broader cardiovascular effects and illuminate diabetes management more than they inform COVID-19 pathology. Their findings align with current 2022 American Diabetes Association/European Association for the Study of Diabetes (ADA/EASD) consensus guidelines for the management of type 2 diabetes mellitus endorsing metformin, SGLT-2 inhibitors, and GLP-1 agonists as first-line therapies, recommending cautious early insulin use, and reserving DPP-4 inhibitors, sulfonylureas, and pioglitazone for selective cases. This is applicable regardless of COVID-19 status. Further research should determine whether infection-related clinical endpoints, such as mortality or hospitalization from COVID-19 or other infections, might serve as valid surrogate markers for cardiovascular outcomes.

## 1 Introduction

Patients with diabetes mellitus are especially prone to developing severe forms of COVID-19 illness and death outcome. The case fatality ratio (CFR) of COVID−19 in the population with diabetes is 7.3%, compared with 2.3% in the general population (1); in addition, they have a 2.8−fold higher risk of admission to the intensive care unit (ICU), a 2.3−fold higher risk of requiring mechanical ventilation, a 2−fold higher risk of developing severe COVID-19, and a 3−fold higher risk of developing cardiovascular (CV) complications (2–4).

It is important to note that the present mini-review focuses specifically on the outpatient, chronic use of antidiabetic medications in patients with pre-existing type 2 diabetes mellitus—the population captured in the observational studies reviewed. The distinct clinical context of inpatient hyperglycemia management, while clinically relevant, falls outside the scope of this review.

At the beginning of the COVID-19 pandemic, certain diabetes mellitus key opinion leaders have warned that special caution should be used while using certain antidiabetic medications during SARS-CoV-2 infection. Numerous observational studies and meta-analyses really did show associations of antidiabetic medications and COVID-19 outcomes—some medications were shown to be protective factors, while others were shown to be risk factors for poor COVID-19 outcomes. Randomized controlled trials (RCTs), which assessed the effect of antidiabetics on COVID-19 outcomes, showed no effect and no clinically relevant anti-inflammatory or immunomodulatory properties of these medications.

The central question of this paper is: “If RCTs showed no significant anti-inflammatory effect, what did observational studies measure?”.

To give our perspective on this question and its answer(s), this paper provides the following:

1. An overview of attitudes toward antidiabetic medications during the COVID-19 pandemic,
2. An overview of the results of observational studies assessing the association between outpatient use of antidiabetic medications and COVID-19 mortality outcome,
3. An overview of the results of large RCTs on antidiabetic medications and COVID-19 outcomes, and
4. An integrated overview and exploration of emerging patterns.

### 1.1 Overview of attitudes toward antidiabetic medications during the COVID-19 pandemic

At the beginning of the COVID-19 pandemic, there were certain fears that use of some antidiabetic medications might lead to unfavorable COVID-19 outcomes. This fear stemmed from the fact that metformin, sodium-glucose cotransporter 2 (SGLT-2) inhibitors, glucagon-like peptide-1 (GLP-1) agonists, and pioglitazone lead to increased expression of the angiotensin-converting enzyme 2 (ACE-2) receptor, the entry site of SARS-CoV-2 virus, at the cell membrane (5, 6). The paper by Borenstein et al., published early in the pandemic, described forming a panel of diabetes experts who wrote initial recommendations for antidiabetic medications use during the COVID-19 pandemic. The panel recommended, in case of acute SARS-CoV-2 infection, to stop metformin, to stop SGLT-2 inhibitors, to observe patients who use GLP-1 agonists, and to continue therapy with dipeptidyl peptidase-4 (DPP-4) inhibitors and insulin (5).

Chen et al. recommended the discontinuation of SGLT-2 inhibitors and the avoidance of the addition of SGLT-2 inhibitors in patients with diabetes with documented or suspected COVID-19 infection. For all other patients with diabetes, they recommended avoiding the addition of SGLT-2 inhibitor before COVID-19 vaccine is available unless the therapeutic benefit clearly outweighs the risk of CV mortality and not to increase previously prescribed SGLT-2 inhibitor dosage (6).

Opposite to these recommendations, Ceriello et al. recommended not amending current antidiabetic therapies regardless of SARS-CoV-2 infection due to favorable CV effects of SGLT-2 inhibitors and GLP-1 agonists, which were deemed especially important by the authors in the context of acute infection (7).

In parallel, papers reviewing antidiabetics’ anti-inflammatory effects and showing great optimism regarding use in COVID-19 were published as well. Some of the papers called antidiabetic medications “a promised land”, “a miracle waiting to happen”, and “savior for COVID-19 patients with diabetes” (8–10). These papers reviewed numerous animal and *in vitro* studies researching anti-inflammatory effects of antidiabetics, dating long before the COVID-19 pandemic, and suggested that antidiabetics could help patients with SARS-CoV-2 infection.

Both the fearful recommendations and the optimistic articles called for clinical trials, primarily RCTs that would show if antidiabetics were a friend or a foe during the COVID-19 pandemic. Recruiting patients for RCT of SGLT-2 inhibitor dapagliflozin started on 20 April 2020 (11) and that for metformin began on 2 June 2020 (12), and their results were published in June 2021 and December 2021, respectively. For about 1 year, observational studies using national data from diabetes registries, hospitals, and insurance companies were the only source of information on the possible clinical effects of antidiabetic medications during SARS-CoV-2 infection.

### 1.2 The results of observational studies

While waiting for RCTs to be conducted and published, many observational studies assessing association of long-term antidiabetics use and COVID-19 outcomes were conducted.

When it comes to the results of these studies, due to such a great quantity of observational studies, we provide an overview of results of 23 of their meta-analyses. These were identified through a PubMed search using the terms “hypoglycemic agents COVID 19 meta-analysis” with filters restricted to English-language studies in humans, conducted in June 2024. Meta-analyses were included if they reported quantitative estimates of the association between pre-admission, outpatient chronic use of antidiabetic medications, and COVID-19 mortality outcomes. The overview is available in **Table 1**.

**Table 1:** Overview of results of 23 meta-analyses of observational studies of associations of outpatient chronic use of antidiabetic medications and COVID-19 mortality.

Antidiabetic medication	Association with COVID-19 mortality Point estimate (95% confidence interval)	Reference
Metformin	OR 0.54 (0.47–0.62)	(13)
	RR 0.60 (0.47–0.77)	(14)
	OR 0.62 (0.50–0.76)	(15)
	OR 0.64 (0.51–0.79)	(16)
	OR 0.64 (0.43–0.97)	(17)
	OR 0.66 (0.58–0.75)	(18)
	ES 0.69 (0.65–0.98)	(19)
	OR 0.69 (0.55–0.86)	(20)
	OR 0.71 (0.50–0.99)	(21)
	OR 0.74 (0.67–0.81)	(22)
	OR 0.78 (0.69–0.88)	(23)
SGLT-2 inhibitors	OR 0.60 (0.40–0.88)	(13)
	OR 0.69 (0.56–0.87)	(24)
	OR 0.80 (0.73–0.88)	(18)
	OR 0.82 (0.76–0.88)	(22)
	Association non-significant	(14)
	Association non-significant	(15)
GLP-1 agonists	OR 0.51 (0.37–0.69)	(13)
	OR 0.53 (0.43–0.66)	(25)
	RR 0.56 (0.42–0.73)	(14)
	OR 0.83 (0.70–0.98)	(18)
	OR 0.91 (0.84–0.98)	(22)
	Association non-significant	(15)
Insulin	OR 1.38 (1.24–1.54)	(22)
	OR 1.52 (1.32–1.75)	(18)
	OR 1.70 (1.33–2.19)	(13)
	OR 2.10 (1.51–2.93)	(26)
	OR 2.14 (1.47–3.10)	(27)
	OR 2.20 (1.34–3.60)	(20)
	OR 2.59 (1.66–4.05)	(28)
DPP-4 inhibitors	OR 0.58 (0.34–0.99)	(26)
	OR 0.75 (0.56–0.99)	(29)
	RR 0.76 (0.60–0.97)	(30)
	OR 0.88 (0.78–1.00)	(22)
	Association non-significant	(31)
	Association non-significant	(13)
	Association non-significant	(14)
	Association non-significant	(32)
	Association non-significant	(20)
	Association non-significant	(33)
	Association non-significant	(15)
	Association non-significant	(18)
Sulfonylureas	OR 0.80 (0.66–0.96)	(20)
	OR 0.93 (0.89–0.98)	(15)
	Association non-significant	(18)
	Association non-significant	(13)
	Association non-significant	(22)
TZD	Association non-significant	(18)
	Association non-significant	(13)
	Association non-significant	(22)
AGI	Association non-significant	(18)
	Association non-significant	(13)

Meta-analyses were found by PubMed search of words *“*hypoglycemic agents COVID 19 meta-analysis” with filters “English” and “humans”, conducted in June 2024. ES, effect size; OR, odds ratio; RR, risk ratio; SGLT-2, sodium-glucose cotransporter 2; GLP-1, glucagon-like peptide-1; DPP-4, dipeptidyl peptidase-4; TZD, thiazolidinediones; AGI, alpha glucosidase inhibitor.

To summarize, the data are entirely consistent for two medications:

- Metformin decreases the risk of COVID-19 mortality (11/11 meta-analyses involving metformin show mortality risk decrease ranging from 22% to 46%).
- Insulin increases the risk of COVID-19 mortality (7/7 meta-analyses involving insulin show mortality risk increase from 1.38 to 2.59 times).

The data are predominantly consistent for two medications:

- SGLT-2 inhibitors probably decrease the risk of COVID-19 mortality (4/6 meta-analyses involving SGLT-2 inhibitors showed mortality risk decrease ranging from 18% to 40%, 2/6 showed non-significant association).
- GLP-1 agonists probably decrease the risk of COVID-19 mortality (5/6 meta-analyses involving GLP-1 agonists show that mortality decrease from 9% to 49% while 1/6 meta-analysis showed non-significant association).

Data are inconsistent for two groups:

- Sulfonylureas: 2/5 meta-analyses involving sulfonylureas show decrease in mortality ranging from 7% to 20%, while 3/5 show non-significant associations.
- DPP-4 inhibitors: 3/12 meta-analyses involving DPP-4 inhibitors show mortality risk decrease ranging from 12% to 42%, 1 shows mortality risk decrease of borderline significance, and 8/12 show non-significant association.

Finally, for two groups, there probably is not enough data to make any conclusions:

- Thiazolidinediones (TZD): 3/3 meta-analyses involving TZD show non-significant association.
- Alpha glucosidase inhibitor (AGI): 2/2 meta-analyses involving AGI show non-significant association.

In summary, meta-analyses of observational studies identified metformin, SGLT-2 inhibitors, and GLP-1 agonists as potentially protective agents against COVID-19 death; insulin as a risk factor for COVID-19 death; uncertainty around DPP-4 inhibitors and sulfonylureas; and insufficient data to draw conclusions regarding TZD and AGIs.

### 1.3 The results of RCTs

Several large, randomized placebo- or standard-of-care-controlled trials were conducted examining the effect of the introduction of metformin, SGLT-2 inhibitor, or pioglitazone following acute infection with SARS-CoV-2 virus. The studies included from 350 to 4,271 patients. The overview of their results is presented in **Table 2**.

**Table 2:** Overview of results of randomized controlled trials assessing the effect of the introduction of antidiabetics in patients with SARS-CoV-2 infection.

Drug	Endpoint	Comparator	No. of participants	Result Point estimate (95% CI) or *N* (%) or median	Reference
Metformin	Hospitalization	Placebo	418	RR 1.14 (0.73–1.81)	(12)
	Viral clearance at day 7			OR 0.99 (0.88–1.11)	
	Fatalities on day 28 (drug vs. placebo)			7 (3.3%) vs. 9 (4.4%), *p* = 0.53	
	Adverse drug reactions			RR 1.02 (0.73–1.45)	
	Clinical improvement on day 28			OR 1.05 (0.71–1.56)	
Metformin	Composite end point (hypoxemia, emergency department visit, hospitalization, or death)	Placebo	1,431	OR 0.84 (0.66–1.09)	(34)
	Hospitalization or death			OR 0.47 (0.20–1.11)	
Dapagliflozin (SGLT-2 inhibitor)	Time to new or worsened organ dysfunction or death	Placebo	1,250	HR 0·80 (0.58–1.10)	(11)
	Change in clinical status by day 30 (recovery)			Win ratio 1.09 (0.97–1.22)	
	Adverse events (drug vs. placebo)			65 (10.6%) vs. 82 (13.3%), *p* not reported	
Empagliflozin (SGLT-2 inhibitor)	28-day mortality	Usual care	4,271	Rate ratio 0.96 (0.82–1.13)	(35)*
	Duration of hospitalization (drug vs. placebo)			Median 8 days vs. median 8 days	
	Composite of invasive mechanical ventilation or death			RR 0.95 (0.84–1.08)	
Dapagliflozin (SGLT-2 inhibitor)	Organ support-free days evaluated through 21 days	Usual care	575	OR 0.74 (0.48–1.13)	(36)*
Pioglitazone	The incidence of a composite outcome composed of (a) the requirement for mechanical ventilation, (b) death, and (c) myocardial damage (drug vs. placebo)	Placebo	350	14 (7.4%) vs. 14 (8.7%), *p* = 0.8	(37)
	Increase in CRP levels [difference between baseline and day 7 (95% CI)], drug vs. placebo			27 (8.6–45) vs. 68 (45–90), *p* = 0.006	

*Recruitment stopped by the independent data monitoring committee due to futility of including further patients. OR, odds ratio; RR, risk ratio; SGLT-2, sodium-glucose cotransporter 2; CRP, C-reactive protein.

To summarize data from **Table 2**, none of the RCTs showed statistically significant differences to placebo for any of the medications compared to placebo, except for pioglitazone in the endpoint of C-reactive protein (CRP) difference on day 7 where patients on pioglitazone had a lower increase of CRP (CRP levels 27 vs. 68 for placebo, *p* = 0.006) (37). However, in clinically relevant endpoints such as mortality, hospitalization, or need for ventilator, there were no differences.

A meta-analysis of the three trials involving SGLT-2 inhibitor RCTs showed no difference in number of deaths on day 28 in comparison to placebo or usual care (OR 0.93, 95% CI 0.79–1.08) (38).

### 1.4 Integrated overview and exploration of emerging patterns

Results of RCTs show that antidiabetics most likely have no clinically meaningful anti-inflammatory effects in the context of acute infective illness. If we consider initial fears previously described with antidiabetics and COVID-19, especially concerning SGLT-2 inhibitors, these results are good news since neither metformin nor SGLT-2 inhibitors showed any reason for concern. Yet, if we consider the optimism stemming from animal and *in vitro* studies, RCT results are disappointing.

## 2 Pitfalls of comparing results of observational studies and RCTs

The first pitfall is that indication bias and confounding are inherent limitations of observational studies. The reasons for prescribing treatment, often related to disease severity or patient characteristics, can confound the association between the drug and outcomes, and despite methods like propensity score matching (PSM) or inverse probability weighting (IPTW), which most of the published observational studies of antidiabetics and COVID-19 outcomes did use diligently, residual confounding often remains unresolved (39).

The second pitfall is that the context and timing of exposure significantly differ between RCTs and observational studies. In RCTs, antidiabetics had not been previously used by the patients but were introduced following the onset of SARS-CoV-2 infection while observational studies analyzed data on patients who had been using antidiabetics for at least several months prior to the onset of SARS-CoV-2 infection. Also, it should be noted that oftentimes, during hospitalization for acute infective disease, oral antidiabetics are discontinued following the onset of a severe acute infective illness; this especially goes for metformin due to fear of lactic acidosis and SGLT-2 inhibitors due to fear of euglycemic ketoacidosis (40).

While these pitfalls warrant careful consideration, the unique value of observational studies in research must not be overlooked. Observational studies do provide valuable knowledge to inform guidelines, decisions, and policy by studying real-world patient populations under routine clinical conditions. They complement RCTs by contributing evidence on diverse patients and clinical settings, often identifying effects not captured in RCTs. They produce results with higher levels of generalizability regarding research participants and practice settings. Data sources frequently used to conduct observational studies can include very large numbers of patients, providing more power than is achieved in most RCTs (41).

## 3 Emerging patterns

If the findings of observational studies of the effects of the antidiabetics in COVID-19 are not due to anti-inflammatory effects of medications, as RCTs have shown, what are they due to instead? Perhaps the findings of these studies are not solely relevant from a COVID-19 standpoint; rather, they should be considered from the standpoint of diabetes mellitus and CV status.

More specifically, we could view them through the lens of antidiabetic medications’ effect on CV outcomes. Prevention of the development of adverse CV outcomes that come with long-standing diabetes mellitus is the main outcome of diabetes management (42).

## 4 Cardiovascular effects of antidiabetics and current clinical guidelines for diabetes management

For all antidiabetics classes, there is extensive body of literature showing that they have some kind of effect on the CV system.

Some of them have positive effects. This is most evident for SGT-2 inhibitors, the class that in addition to indication of diabetes mellitus type 2 also has approved indication for the treatment of heart failure and chronic kidney disease (43). These approvals were of course gained following extensive clinical trials finding SGLT-2 inhibitors beneficial for major adverse cardiovascular events (MACE), and heart failure MACE includes CV death, non-fatal myocardial infarction, and non-fatal stroke (44).

In the US, the FDA has additionally approved GLP-1 agonists for reducing the risk of MACE in adults with established CV disease and obesity or overweight (45). Davies et al. find GLP-1 agonists to be beneficial for MACE and neutral toward heart failure (42).

Metformin has been used since the 1960s in the management of diabetes mellitus, Davies et al. find it possibly beneficial for MACE and neutral toward heart failure (42). There is an extensive body of evidence that they are beneficial for MACE (46, 47). There is even a study showing that metformin could possibly be protective of heart failure development (48).

Insulin, on the other hand, probably has negative effects on MACE, even though Davies et al. describe them as MACE-neutral (42). Literature search finds a lively discussion on this topic, and the results of many large observational studies seem far from “neutral”. This is reviewed extensively, namely, by Kolb et al. and Nolan et al., and describing this would be beyond the scope of this paper (49, 50). There are theories on how insulin could negatively affect the CV system—hypoglycemia is an independent risk factor for many adverse CV outcomes (51–57), insulin is related to endothelial dysfunction (58, 59), insulin is related to reduced response to antiplatelets (60), insulin is related to changes in fatty acid metabolism in cardiac muscle cells (61), some studies find insulin to have proatherogenic effects (62), and insulin is associated with weight gain (63). It seems that the many benefits of insulin in patients with diabetes mellitus come from the prevention of microvascular (and not macrovascular) complications (64). When it comes to heart failure, Davies et al. see insulin as neutral.

When it comes to DPP-4 inhibitors, sulfonylureas, and TZD, CV effects are less clear.

In their Summary of Product Characteristics (SmPCs) list warnings, DPP-4 inhibitors alogliptin and saxagliptin could be related to heart failure; this is based on a large RCT for saxagliptin (44, 65) and from *post-hoc* analysis of RCT for alogliptin (66, 67). Davies et al. see them as MACE-neutral, but as potentially risky for heart failure. When it comes to MACE, Davies et al. see them as neutral, which is supported by a meta-analysis of RCTs where no association to CV risks is shown (68).

Davies et al. see sulfonylureas as both MACE- and heart failure-neutral; however, SmPC of gliquidone lists reported the adverse reactions of CV insufficiency, angina pectoris, and extrasystoles (69). Literature search finds observational studies that imply the association of the drug and poor CV outcomes (51, 52, 70–72). A randomized study showed that sulfonylurea in comparison to metformin caused more CV events without death outcome and death of any outcome (73). A meta-analysis of observational and randomized trials showed increased risk of death from CV causes and of a composite outcome (74).

Davies et al. see TZD as potentially beneficial for MACE but a risk factor for heart failure. However, there are studies that show the potential association between pioglitazone and ischemic heart disease (75).

To summarize, metformin, SGLT-2 inhibitors, and GLP-1 agonists have beneficial CV effects while insulin seems to affect the CV system adversely in terms of increased risk of MACE. For DPP-4 inhibitors, sulfonylurea, and pioglitazone, CV effects are less clear.

Clinical guidelines by Davies et al. put metformin as a central antidiabetic medication, although they avoid the wording “first line”, but also note that SGLT-2 inhibitors and GLP-1 agonists could, in the future, become first line in certain subpopulations of patients with diabetes mellitus and encourage their early introduction. When it comes to sulfonylurea, DPP-4 inhibitors, and pioglitazone, they mention them more as additions when glycemic goals are not achieved or if patients have hepatic comorbidities (pioglitazone). For insulin, they seem careful and do not encourage rushing to introduce it, although they do warn against clinical inertia, too (42).

We should remember that COVID-19 also has CV effects.

## 5 COVID-19 and the cardiovascular system

During acute infective illness, the CV system is strained. Severe COVID-19 illness can lead to CV disorders, even in individuals without previously diagnosed CV diseases, which may manifest as myocardial injury, acute coronary syndrome (ACS), heart failure, arrhythmia, and coagulation disorders (76–83).

The incidence of acute myocardial injury in patients with severe COVID-19 without prior CV disease ranges from 7% to 12%, rising to 22% in ICU admissions (1, 77, 78). ACS has a reported incidence of 45% in ICU-treated and 6% in non-ICU hospitalized patients with COVID-19 (80). Heart failure affects approximately 24% of all patients and 49% of those who died (81). Cardiac arrhythmias occur in approximately 17% of patients with severe COVID-19, increasing to 44% among ICU cases (78). Coagulopathy increases death risk 18-fold and manifests as venous thromboembolism, pulmonary embolism, and arterial thrombosis, affecting 35.3% of ICU patients and 2.6% of ward patients (76, 80, 84).

The pathophysiology of CV complications in severe COVID-19 is not fully understood (76, 82). Possible mechanisms include direct viral cytotoxicity to cardiomyocytes via ACE-2 receptors, endothelial dysfunction in coronary microvessels, and systemic inflammation causing plaque rupture, coronary spasm, and microthrombi formation (76, 82, 85). Heart failure may arise partly due to increased cardiac workload from acute lung injury (76, 82). The mechanisms underlying hypercoagulability are possibly severe inflammatory responses and endothelial dysfunction (76).

## 6 Synthesis

It seems that antidiabetics which have positive effects on CV system (metformin, SGLT-2 inhibitors, GLP-1 agonists) are also protective of COVID-19 death when used in chronic outpatient therapy of DM type 2. Insulin is shown to be a risk factor for COVID-19 death when used in chronic outpatient therapy for DM type 2. For chronic outpatient use of DPP-4 inhibitors, sulfonylurea, and pioglitazone, there seems to be a lack of clarity when it comes to both CV outcomes and COVID-19 mortality.

We said that the central question of this paper was “If RCTs showed no significant anti-inflammatory effect, what did observational studies measure?”; however, now following all the presented information, we can further specify the question to “Do observational studies on the association of antidiabetics and COVID-19 outcomes provide information on COVID-19 or on diabetes mellitus?”.

In our view, based on all information available and presented in this paper, these studies offer limited insight into COVID-19 itself but valuable information about the management of type 2 diabetes. It seems plausible that, if antidiabetic medications do not exert clinically significant anti-inflammatory effects, and this is suggested by RCT findings, then the observational studies have in fact revealed how these medications influence diabetes mellitus or, to be more precise, the CV system.

We suggest that in all these observational studies, COVID-19 outcomes unintentionally served as surrogates of CV outcomes. There was an intention to study COVID-19 and to learn more about it; however, we believe that this did not happen in observational studies, but instead we just got confirmation of what is already known about CV effects of antidiabetics. COVID-19 outcomes were just surrogates of CV outcomes.

## 7 Conclusion

If the results of observational studies on the association between chronic outpatient use of antidiabetic medications and COVID-19 outcomes are interpreted as more informative about diabetes mellitus management than about COVID-19 pathology, then their findings can be seen as supporting the recommendations of Davies et al. for the management of type 2 diabetes mellitus. Metformin, SGLT-2 inhibitors, and GLP-1 agonists should be the preferred first-line medications; DPP-4 inhibitors, sulfonylureas, and pioglitazone should be reserved for specific situations when additional glycemic control is necessary; and insulin should be introduced thoughtfully, without rushing. These principles apply regardless of SARS-CoV-2 infection status.

It is warranted that future investigations evaluate whether infection-related clinical endpoints, such as mortality/hospitalization due to COVID-19 or other infectious diseases, may serve as valid surrogate markers for CV outcomes.
